# Evaluation of Percutaneous Coronary Intervention and stenting of Left Main Coronary Artery Stenosis in Tehran’s Rajaie and Lavasani Hospitals from 2010 to 2011

**DOI:** 10.5812/cardiovascmed.12594

**Published:** 2013-10-28

**Authors:** Safarali Abdolrahimi, Hamidreza Sanati, Alireza Fatahian

**Affiliations:** 1 Cardiovascular Intervention Research Center, Rajaie Cardiovascular Medical and Research Center, Iran University of Medical Sciences, Tehran, IR Iran

**Keywords:** Percutaneous Coronary Intervention, Coronary Artery Disease, Myocardial Revascularization

## Abstract

**Background:**

Data of the results from treatment of unprotected and protected LMCA diseases with PCI and stent implantations in our country were limited. Surgical therapy is considered as an standard care for patients with unprotected LMCA stenosis. This notion is based on some randomized and observational studies performed three decades ago which convincingly showed superiority of CABGs over medical therapy. Moreover, preliminary studies have shown that the use of DES for the treatment of unprotected LMCA diseases is associated with very favorable mid-term outcome, which is highly competitive with that of surgery, especially for ostial lesions

**Objectives:**

This study sought to evaluate one year safety and effectiveness of PCI and stenting in LMCA disease.

**Patients and Methods:**

We performed a one year clinical follow-up of any patients with LMCA disease “Protected and Unprotected” who underwent PCI and stenting (n = 40) with BMS (n = 17) or DES (n = 23) in Tehran’s Rajaje and Lavasani hospitals from September 2010 to September 2011. The primary end points were all-cause mortality, and MACCE which consisted of the composite of death, MI, stroke, and target vessel revascularization, and the duration of hospitalization change the severity of angina pain and the function class of physical activity.

**Results:**

In the one year follow-up, the adjusted risk of death was 5% and the composite of death, MI, stroke and target vessel revascularization (MACCE) was 22%. In 94.7% the number of patients, the severity of angina pain were decreased, and in 92.5% of patients, the function class of physical activity has been improved. The duration of hospitalization was 4.38 ± 1.63 days which was less than that of CABGs.

**Conclusions:**

For the treatment of protected and unprotected LMCA diseases, PCI with stent implantation is effective, and leads to decreasing the mortality and the death rate, MI, stroke, the severity of angina pain, and improving the function class of physical activity and tolerance.

## 1. Background

Left main coronary artery disease (LMCAD) is found in 4% to 6% of all patients undergoing coronary angiography (1, 2). The prognosis of LMCAD is poor. In fact, unprotected left main CAD carries the worst prognosis compared with single-, double-, or triple-vessel disease, probably because it is frequently associated with severe multi-vessel disease and an extensive amount of jeopardized myocardium. In the 1970s, studies showed that without revascularization, the percentage of survival of patients with more than 50 % left main stenosis is 66% at three years (3). The survival is worse with higher grade lesions. Existing more than 70% of left main stenosis, only 41% of patients survive after three years. These numbers underscore the profound importance of the LMCAD, and the potential benefit that might be achieved by treating patients with left main stenosis. Coronary Artery Bypass Grafting (CABG) surgery is considered as the standard care for the treatment of Unprotected LMCAD (4). This notion is based on some randomized and observational studies performed three decade ago, which showed that surgery is associated with significant improvement in survival compared with medical therapy (5, 6). Continued technical evolution of percutaneous coronary intervention (PCI), including the recent introduction of Drug Eluting Stents (7-9) (DES) and aggressive antiplatelet therapy (10, 11), has renewed the interest for the percutaneous treatment of ULMCA stenosis (12-16).

## 2. Objectives

The objective of this study was to evaluate one year safety and effectiveness of PCI and Stenting in LMCAD.

## 3. Patients and Methods

### 3.1. Study Protocol and Patient Population

In this descriptive case series study, we performed a one year clinical follow up of any patients with left main Coronary Artery Disease (LMCAD), either protected (with history of previous CABGs) or unprotected (without history of previous CABGs), who underwent PCI and stenting in Rajaie Cardiovascular center and Lavasani cardiovascular hospital, Tehran, IR Iran, over a period of one year from September 2010 to September 2011. We excluded patients except those with atherosclerotic disease of LMCA for example left main coronary artery dissection and comorbidities as well as Valvular Heart Disease, malignancies, renal insufficiency, pulmonary or hepatic failure. A total forty consecutive subjects (31 men and 9 women) were recruited. 

### 3.2. Statistical Analysis

The data were recorded in SPSS 15 for windows (SPSS Inc. Chicago, IL, USA). This analysis was used to identify and evaluate the outcome of these patients and compare the risk factors between them. Continuous variables are presented as mean ± SD. The student`s t-test was applied to compare the data between two groups with a normal distribution. Otherwise, a non-parametric Mann-Whitney U test was used. A P value less than 0.05 was considered statistically significant. Our database contained detailed information on patients’ demographics, pre-procedural risk factors, procedure details, post-procedure hospital course, mortality, and MACCE (Major Adverse Cardiac and Cerebrovascular Events which consist of Death, MI, CVA, & Target vessel revascularization) outcomes. These data consist of gender, age, the history of previous CABGs, diabetes, hypertension, dyslipidemia, smoking, familial history of CAD ,as well as post procedural MI ,CVA, target vessel revascularization, the duration of hospitalization, changes in the severity of angina pain, and changes in the function class of physical activity.

## 4. Results

### 4.1. Patient Characteristics

Study population consists of 40 post- PCI and Stenting on LMCAD patients with one year follow-up. The Mean age during the procedure was 62.8 ± 10.8 years from which 77.5% was men, and 85% had Protected LMCA .Diabetes was manifested in 37.5%, HTN in 35%, Dyslipidemia in 27.5%; 30% were smoker, and 5% had family history of CAD. The site of lesion in LMCA was 35% in proximal portion, 5% in mid portion, and 60% in distal portion.

### 4.2. Baseline Characteristic of Patients with & Without Post- procedural MACCE 

Demographic and clinical characteristic of the study population by MACCE were summarized in Table 1. 

**Table 1.** Demographic, Clinical, Angiographic Characteristics by post procedure MACCE

	Major Adverse Cardiac and Cerebrovascular Events		P value
	Yes (n = 9)	No (n = 31)	
**Age, y, Mean ± SD**	64.1 ± 8	62.41 ± 10	0.001
**Male** ^a^	66.7	80.6	0.377
**History of CABGs**	77.8	87.09	0.491
**DM**	77.8	25.80	0.24
**HTN**	33.3	35.48	0.674
**Dyslipidemia**	22.2	29.03	0.656
**Smoking**	44.4	25.8	0.283
**Positive of family history of CAD**	22.2	0	0.007
**DES**	44.4	61.3	0.368
**Distal lesion**	66.7	58.1	0.713

^a^ The data are shown with percentage (%)

In this study, the total incidence of MACCE after one year follow-up was 22% (Table 2). 

**Table 2.** Types of MACCE in this Study

Types of MACCE	No. (%)
**Death**	2 (22.2)
**MI**	4 (44.4)
**CVA**	1 (11.2)
**Revascularization**	2 (22.2)

Patients with MACCE were older than patients without MACCE (64 ± 8 vs. 62.4 ± 10, P = 0.001). The prevalence of comorbidities including diabetes (77.8% vs. 25.8%, P = 0.24). Smoking (44.4% vs. 25.8%, P = 0.285), family history of CAD (22.2% vs. P = 0.007) were significantly higher in patients with MACCE; however the prevalence of hypertension, dyslipidemia were slightly lower in MACCE group. The prevalence of MACCE based on its type was: MI: 4 (10%), Death: 2 (5%), Revascularization: 2 (5%), and CVA: 1 (2.5%). Patients with MACCE had lower history of CABGs (77.8% vs. 87.09%, P = 0.377), and 44.4% 0f patients with MACCE had drug eluting stents (DES) and the others had bared metal stents (BMS). On the other hand, 17.39% of patients with DES had affected by MACCE, but 29.4% of patients with BMS were too. Therefore, greater percent of patients had affected MACCE by BMS in comparison with DES (P = 0.368). More patients of MACCE group had distal lesion in LMCA (66.7%, distal lesion vs. 33.3%, proximal lesion, P = 0.368). Both patients who died in this study were male, diabetic, with positive family history of coronary artery disease, and had not history of CABGs in the past (Unprotected) . In this study, four patients had MI (NSTEMI), and one patient had CVA. Two patients in our study had undergone the recurrent revascularization (one CABG, & the other PCI with Stenting) .The average time of hospitalization was 4.38 ± 1.63 days in this study. In our study, the severity of angina pain decreased in 94.7% of patients, and the function class of physical activity improved in 92.5% of them. Of all patients in this study, only one stent was used for PCI and Stenting of LMCA. The stents were drug eluting (DES) in 57.5%, and bare metal (BMS) in 42.5% of patients. The length and width of stents were 18.45 ± 5.6 mm and 3.14 ± 0.38 mm respectively. 

## 5. Discussion

Surgical therapy is considered as the standard care for patients with unprotected LMCA stenosis. This notion is based on some randomized and observational studies performed three decades ago that convincingly showed superiority of CABGs, over medical therapy. Moreover preliminary studies have shown that the use of DES for the treatment of unprotected LMCA disease is associated with very favorable mid-term outcome, which is highly competitive with that of surgery, especially for ostial lesions (12, 13, 15, 16). In addition, three recently published observational studies comparing CABGs vs. DES for the treatment of unprotected LMCA disease have shown similar rates of mortality at mid-term follow-up (17-19). The present study showed that PCI and Stenting of LMCA is an effective treatment in good selected cases of these patients. This treatment not only improves short and midterm prognosis but also can relieve symptoms, improve the function class of patients activity and decrease the duration of hospitalization compared with CABGs. In this study, like what was proved in the previous studies, the prevalence of MACCE was greater in unprotected LMCAD patients, and was higher in patients with bare metal stents (BMS) than drug eluting stents (DES). In our study, diabetes and positive family history of CAD were two important risk factors of mortality, whereas smoking was the most important risk factor for nonfatal myocardial infarction. In this study, like what was found in the previous studies, the most common site of the left main coronary artery lesion was in distal portion.
